# *Philodryas* (Serpentes: Dipsadidae) Envenomation, A Neglected Issue in Chile

**DOI:** 10.3390/toxins11120697

**Published:** 2019-11-29

**Authors:** Félix A. Urra, Alejandro Bruno Miranda-Calle, Ramiro Araya-Maturana

**Affiliations:** 1 Programa de Farmacología Molecular y Clínica, Instituto de Ciencias Biomédicas (ICBM), Facultad de Medicina, Universidad de Chile, Independencia 1027, Casilla 7, Santiago 7800003, Chile; 2 Network for Snake Venom Research and Drug Discovery, Santiago 7800003, Chile; raraya@utalca.cl; 3 Área de Herpetología, Colección Boliviana de Fauna, c. 27 de Cota Cota, La Paz 10077, Bolivia; abrunomirandac@gmail.com; 4 Instituto de Química de Recursos Naturales, Universidad de Talca, Casilla 747, Talca 3460000, Chile; 5 Programa de Investigación Asociativa en Cáncer Gástrico, Universidad de Talca, Casilla 747, Talca 3460000, Chile

**Keywords:** snakebite, opisthoglyphous, *Philodryas*, toxins, colubrid, therapeutics

## Abstract

Snakebite envenomation is considered a neglected tropical disease, although it also occurs outside the tropics. In this work, we analyzed the literature on *Philodryas* species in Chile (*Philodryas chamissonis*, *P. simonsii*, and *P. tachymenoides*) from 1834 to 2019, searching for epidemiological, clinical, and molecular aspects of envenomation. Ninety-one percent of the studies found regarded taxonomy, ecology, and natural history, suggesting that snakebites and venom toxins are a neglected issue in Chile. All snakebite cases reported and toxicological studies concerned the species *Philodryas chamissonis*. Using 185 distributional records from the literature and museum collections for this species, we show for the first time that the reported snakebite cases correlate with human population density, occurring in the Valparaiso and Metropolitan regions in Central Chile. The reduced number of snakebite cases, which were previously considered as having a low incidence in Chile, may be a consequence of under-reported cases, probably due to the inadequate publication or scarce research on this issue. Absence of information about official pharmacological treatment, post-envenoming sequels, clinical management of particular patient groups (e.g., with non-communicable diseases, pregnant women, and the elderly) was also detected. In conclusion, despite having over 185 years of literature on Chilean snakes, knowledge on the envenomation of *Philodryas* genus remains scarce, seriously affecting adequate medical handling during an ophidic accident. This review highlights the need to develop deep research in this area and urgent improvements to the management of this disease in Chile.

## 1. Introduction

Snakebite envenomation is considered a neglected tropical disease (NTD) that also occurs outside of the tropics [1,2] and requires local attention in each country to understand the disease’s epidemiology in order to improve the effectiveness of treatments and to contribute to reduced deaths and disabilities from a medical perspective [3]. The World Health Organization (WHO) has stated that the control and documentation of snakebite envenoming has long been hampered by poor-quality epidemiological data and poor investment in the study of toxicology beyond the ecological aspects of snakes [4,5].

The genus *Philodryas* (Wagler 1830) is currently composed of 23 rear-fanged snake species widely distributed in South America [6,7,8,9]. Most species inhabit the lowlands of cis-Andean South America, while only four occur along the trans-Andean zones in Chile, Peru, and Ecuador [6,8].

These species are generally considered harmless for humans; however, abundant data on human snakebites with medical significance [10] and their epidemiological implications are available for the cis-Andean species group, especially for *P. baroni* [11], *P. olfersii* [12,13,14,15], *P. patagoniensis* [13,16,17], and *P. viridissima* [18,19]. The clinical manifestation of envenoming by some *Philodryas* species, such as local pain, swelling, erythema, ecchymosis, and regional lymphadenopathy, resemble the local symptoms of *Bothrops* bites [20,21], and *P. olfersii* and *P. patagoniensis* venoms exhibit immunological cross-reactivities to anti-*Bothrops* sp. serum [21]. Moreover, the components of cis-Andean *Philodryas* venom secretions have been studied using transcriptomic and biochemical approaches [22], identifying at least five classes of toxins involved in the main clinical effects observed in bitten humans, such as snake venom metalloproteases (SVMPs), snake venom serineproteases (SVSP), cysteine-rich secretory proteins (CRISPs), C-type lectin proteins (CLPs), and natriuretic peptides (NPs) [23,24,25,26,27,28]. All this contrasts with the scarce toxicological information of the species of trans-Andean group *P. amaru*, *P. chamissonis*, *P. simonsii*, and *P. tachymenoides* [29].

**Figure 1:**
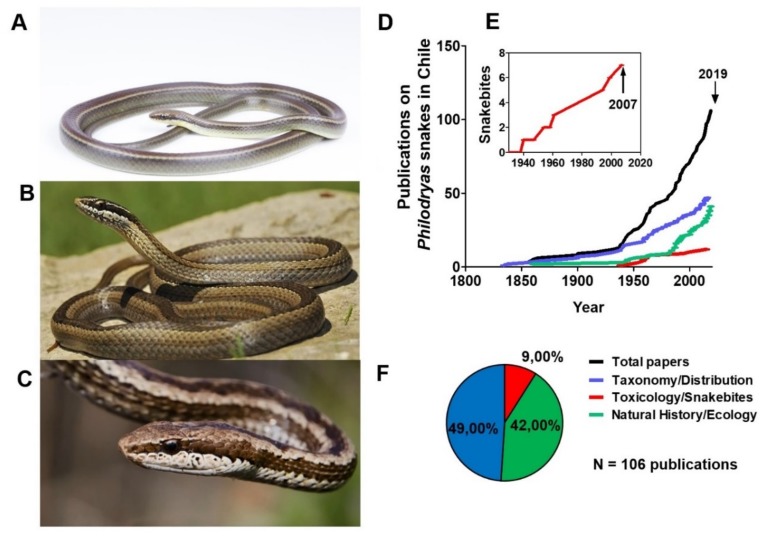
Species of the *Philodryas* genus recognized for Chile. (**A**) *Philodryas simonsii*, referential photography, specimen QCAZR13878, Pontifical Catholic University of Ecuador Collection; (**B**) *Philodryas chamissonis* from Maule Region, Chile; (**C**) *Philodryas tachymenoides* from Arica and Parinacota Region, Chile. (**D**) The articles published on snakes in Chile covering taxonomic/geographical distribution (blue line), toxicology/human snakebite reports (red line), and natural history/ecology (green line). Total compiled works: 106. (**E**) The cumulative total number of reports on ophidism by *Philodryas* species and the (**F**) percentage of articles published on *Philodryas* snakes present in Chile.

Although evidence concerning snake envenoming caused by Chilean snake species of the genus *Philodryas* exists from the 1940s, poorly available information on snakebite envenoming situates Chile within countries with insufficient clinical and epidemiological information [30]. Moreover, the apparent low incidence of snakebites and lack of human mortality associated to snake envenoming [31,32] have led to the underestimation of morbidity and non-inclusion in official health programs [33]. Certainly, this knowledge gap highlights the need for identifying the research focus that requires important contributions for new treatments and effective diagnosis as well as molecular and preclinical information. In this work, the literature from 1834 to 2019 on Chilean *Philodryas* species *P. chamissonis*, *P. simonsii*, and *P. tachymenoides* (Figure 1A–C) [34] was reviewed and analyzed for the first time, revealing that snakebites and venom toxins are neglected issues by researchers in Chile.

## 2. Results and Discussion

A total of 106 articles about *Philodryas* species in Chile were reviewed for the period 1834–2019 (185 years, Table S1). As detailed in Figure 1D,E, the period 1938–2007 concentrates all the snakebite case reports on Chilean snakes, showing that the first formal report published on *Philodryas* snakes was in 1940 [35]. Nine percent of the studies focused on snakebite reports or toxicological aspects of venom; 42% on natural history and ecology; and 49% on the distribution and taxonomy of these species (Figure 1F). The obtained data suggest historic biases in the studies on the *Philodryas* genus, suggesting snakebites and venom toxins are neglected issues in Chile.

### 2.1. Epidemiological Aspects

The literature, from 1834 to date, shows that all snakebite cases reported for the *Philodryas* genus in Chile (seven demonstrable cases in total) belonged to *Philodryas chamissonis*. For *P. simonsii* and *P. tachymenoides*, no reported cases of biting and envenoming in humans were found.

**Figure 2:**
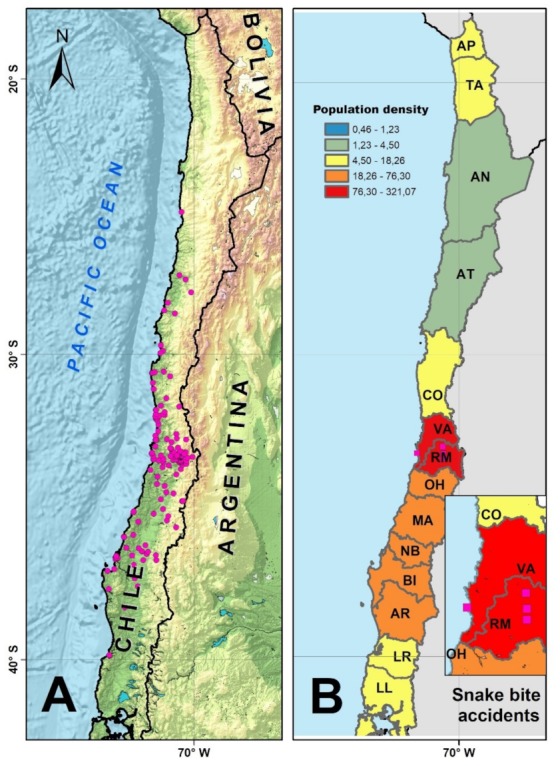
Distribution of *Philodryas chamissonis* and the location of snakebite reports for Chile. (**A**) *P. chamissonis* distribution in Chile obtained from the literature and museum records shown in circles. (**B**) Human population density, inhabitants per square kilometer, in the different regions of Chile (abbreviated denominations according to ISO 3166-2). Reported cases of snakebite accidents in the Valparaíso and the Metropolitan regions are shown in squares.

We obtained 185 distributional records from the literature and museum collections for *P. chamissonis* (Table S2), and we studied if the reported snakebite cases correlated with human population density. As Figure 2 shows, reported snakebite accidents may be related to a greater human contact (high population density) as shown by the greater number of *P. chamissonis* records, especially in the Valparaíso and Metropolitan regions (Figure 2). We did not rule out the occurrence of unreported snakebite accidents throughout its 25° to 40° south latitude distribution, from Los Ríos to Tarapacá regions in Chile. 

All snakebites produced by *P. chamissonis* were due to the fact of improper animal manipulation or casual snakebites in the upper extremities, especially the fingers. The age of the victims ranged from 11 to 25 years; six cases involved men and one case a woman [31,36,37,38]. All accidents occurred during the summer season, in December and January. Interestingly, similar epidemiological observations have been reported for patients with ophidism by *P. patagoniensis* and *P. olfersii* in Brazil [14,17]. Notably, a non-scientific paper mentions a case of human death produced by *P. chamissonis* envenoming [6]; however, this citation is a wrong translation from an original paper in Spanish published by Gigoux [35], which states: “…una persona mordida”, a bitten person and not a dead person (“…una persona muerta”). Consistent with previous observations [38], our literature analysis did not find evidence of *Philodryas* envenoming-caused death in Chile.

### 2.2. Clinical Aspects and Pharmacological Treatment

Front-fanged species of the *Crotalus*, *Bothrops*, *Lachesis*, and *Micrurus* genera are responsible for the majority of snakebite-related human deaths in South America [32,39,40,41]. Despite this, clinical manifestations of envenoming by *Philodryas* resemble the symptoms produced by front-fanged species, as *Bothrops* spp. [21], suggesting an adoption of a more careful evaluation of the victims and its medical treatment [13]. In contrast to other South American countries, there are only rear-fanged snakes of the *Pseudalsophis*, *Tachymenis*, and *Philodryas* genera in Chile. From all they, *P. chamissonis* snakebite reports with clinical importance have been published describing the evolution of symptoms. At the time of the bite, all patients did not report pain [31,38]. Local lesions were made up of small punctiform wounds produced by snake teeth [42] which, in some cases, remained in the bite site [38]. The reported cases recorded inflammation at the inoculation site in the first 10 to 30 min, accompanied by an intense throbbing pain and heat sensation spreading throughout the affected limb in the following hours [36,38]. Laboratory tests for pediatric and adult patients showed hypoprothrombinemia and prolonged activated partial thromboplastin time (aPTT) with liver, urine, electrocardiogram, plasma electrolyte, and blood glucose tests in normal ranges within the first 24 h after ophidic accident [16,31,36]. The local symptoms produced by cis-Andean *Philodryas* species, such as *P. olfersii*, *P. patagoniensis*, *P. viridissima*, and *P. baroni*, are similar to those described for *P. chamissonis*, i.e., edema, intense pain, bleeding for few minutes, swelling, ecchymosis, and, in some cases, lymphadenopathy; the duration of these effects ranged from 1 to 7 days, with positive clinical results [11,12,14,17,18].

During this time, edema with a stiff appearance induced by *P. chamissonis* snakebite extended to the shoulder region and chest area with ecchymosis spots and supra-epitrochlear and axillary adenopathies sensitive to palpation [16,31,36]. Some patients exhibited fever, headache, nausea, and a tendency to hypotension. There were no available data about specific or official pharmacological treatment for snakebite patients; instead, empiric treatments consisted of the use of corticosteroids (betamethasone), intravenous antihistamines, and antibiotics (cloxacillin + gentamicin or ampicillin) that reduced edema, headache, nausea and fever, without affecting pain. This treatment caused the regression of the ophidism at 3–4 days. After 7–10 days, ecchymosis and residual inflammation [36] were observed, affecting movement (e.g., writing) with slight pain [38]. Some recommendations of *P. chamissonis* snakebite have been proposed by Neira et al. [31] such as immobilizing the bite limb, avoiding the use of tourniquets or suction of the wound, washing with warm soapy water immediately, and followed with clinical management for hydration, analgesia, and systemic corticosteroids [31]. 

### 2.3. Toxicological Aspects: Pre-Clinical and Molecular Evidence

We found three published reports on experimental toxicity in animals or toxin studies for *Philodryas* species in Chile, representing the 3% of the analyzed literature which only involved *Philodryas chamissonis* [29,43,44]. Studies conducted to understanding the effect of the *P. tachymenoides* (*Dromicus tachymenoides*) and *P. simonsii* bites in guinea pigs showed uncertain results [44], because data was not detailed in any publication; therefore, no work in toxicology for both species has been reported (Table 1). The envenoming gland is white with small granulations that connect with the posterior teeth [44]. These are located behind the posterior edge of the eye, are moderately curved, and have a partially closed venom-conducing canal [45]. Extensive details on cranial osteology can be found in Habit et al. [46]. The venom secreted from the envenoming gland has been described as having a milky-white appearance [44]. Although a size-related shift in the dietary habits of neonates and adult *P. chamissonis* has been reported [47,48], ontogenetic variations in the dental apparatus and venom gland morphology are still unknown. 

Even though the toxicological effects of venom of several *Philodryas* species have been extensively studied in animal models, such as hematological alterations [4]; edema [49], myotoxicity [50], and neurotoxicity [51], the effects of *P. chamissonis* venom in vivo have been poorly explored. In *Mus musculus* mice, the 2.5 mg intraperitoneal inoculation of venom gland extract of *P. chamissonis* produces dyspnea, decay, ataxia, and hind limb paralysis, producing death at 115 min, but minor doses do not cause death [43,44]. These animals present lesions with hemorrhages at the inoculation site, compromising the abdominal muscles and affecting the small intestine, mesentery, pleural cavity, spleen, and kidneys with hemorrhagic infiltrations [43,44]. Consistent with this, the direct *P. chamissonis* inoculation in a mouse causes damage in the peritoneum with incipient hemorrhagic sites [29]. In muscle, bites produce hemorrhage with evident edema, accompanied by small coagulopathies in the subcutaneous tissue [29]. This evidence has been related with the presence of toxins with protease, anti-coagulant, and pro-inflammatory action [29].

From studies on venom of cis-Andean *Philodryas* species, it has been recognized that it presents high proteolytic and hemorrhagic activities but lack of esterase [52], nucleotidase/DNAase [53] and phospholipase A2 activities [21,54] Consistent with this, biochemical characterizations of the venom using extracts from dialyzed and vacuum-dried parotid glands of *P. chamissonis* suggest a high dose-dependent proteolytic activity in vitro. These extracts complete the gelatin proteolysis in 3 h and lack hemolytic or coagulant effects at 24 h of exposure [43]. In line with this, molecular identification of five toxin-encoding genes [29] which are widely described in snakes of the families Elapidae and Viperidae [22,55,56] have been reported: snake venom metalloprotease type-III (SVMP), snake venom serine protease (SVSP), cysteine-rich secretory protein (CRISP), C-type lectin-like protein (α and β CLP), and natriuretic peptide (NP) [29].

**Figure 3:**
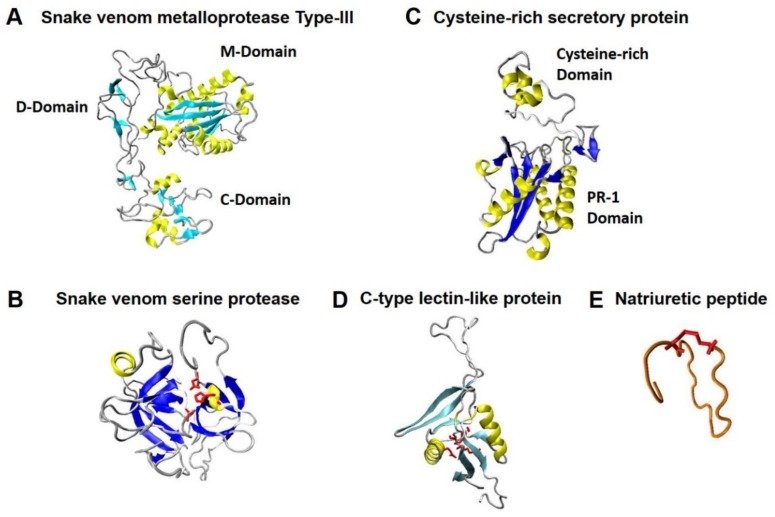
Predicted 3D structures for *P. chamissonis* toxins. (**A**,**B**) Putative toxins with enzymatic activities and (**C**–**E**) putative catalytic activity-lacking toxins with ligand properties. Representations from Urra et al. [29].

The SVMP-Pch (Figure 3A) is a predicted single polypeptide of 615 amino acids that exhibits a disintegrin-like domain with an aspartic–cysteine–aspartic (DCD) motif and a cysteine-rich domain similar to the sequences reported for *P. olfersii* [24] which may have α-fibrinogenolytic, hemorrhagic, and pro-inflammatory activities as patagonfibrase, a SVMP isolated from *P. patagoniensis* venom [26,58]. In addition, SVSP-Pch (Figure 3B) is a putative peptide of 261 amino acids that includes a signal peptide, an activation peptide sequence, and a native serine protease of 226 amino acids with an extension of cysteine residue-containing C-terminal region. In Figure 3B, conserved residues H74, D119, and S213 that compose the catalytic site are shown in red. The SVSP-Pch exhibits three putative N-glycosylation sites (^112^Asn-Cys-Thr^114^, ^126^Asn-Ser-Ser^128^, and ^130^Asn-Asn-Ser^132^) that may contribute to the enzyme stability and its macromolecular selectivity, similar to other SVSPs [59,60]. On the other hand, the non-enzymatic CRISP toxins from front-fanged snakes induces depolarization-induced concentration of arterial smooth muscle of rat tail by blocking cyclic nucleotide-gated channels, voltage-gated Ca^2+^ channels, voltage-gated K^+^ channels, and Ca^2+^-activated K^+^ channels activities [61,62,63]. The molecular characterization of CRISP-Pch (Figure 3C) reveals that this toxin lacks all the putative binding sites and domains required for the inhibition of ionic channels, suggesting that CRISP-Pch may have different biological effects than those produced by CRISP from front-fanged snakes [29]. Consistent with this observation, the predicted amino acid sequence for CRISP-Pch has matched with fragments of the N-terminal and PR-1 domain of patagonin, a CRISP toxin isolated from *P. patagoniensis* venom with unusual myotoxic activity [28]. 

The C-type lectin-like proteins are known from front-fanged snake venoms, affecting the blood coagulation and the platelet aggregation [64,65]. C-type lectin-like protein-Pch is a putative α/β heterodimeric CLP lacking calcium-binding sites, of which its biological effect remains unknown [29]. Figure 3D details the amino acidic residues of the β-subunit (Ser66, Leu68, Glu72, and Lys159) that do not form the Ca^2+^-binding site. Natriuretic peptides are present in the venom of Viperidae and Elapidae species and have potent hypotensive effects [66]. The predicted peptide NP-Pch exhibits the common ring of the vasoactive natriuretic peptides constituted by 17 amino acids (CFGX_12_GC motif), bridged by an intra-molecular disulfide bond by Cys-265 and Cys-281 [29] (Figure 3E, disulfide bond is shown in red) which is required to interact with membrane-bound receptors with guanylyl cyclase activity [67].

In addition, taking into account that the toxins responsible for causing inflammation and initial hemorrhage, such as SVMP type-III and SVSP described for *P. chamissonis* [29], are evolutionarily conserved, and being present in other species of the genus, such as *P. patagoniensis* [26,27,58], *P. olfersii* [24,51,68], *P. baroni* [69], it is possible that the venom of the remaining trans-Andean species of *Philodryas* also have these toxins and the same activities.

### 2.4. Systemic Symptoms and Dry Bites

In *Philodryas* snakes, systemic envenoming is infrequent; however, there exists a case of 2 year old child bitten in the finger by *P. olfersii* that presented the common local effects (local pain, ecchymosis, transient bleeding) with abdominal pain and vomiting [14]. Interestingly, Peichoto et al. [70] reported the local effects of a bite by *P. olfersii latirostris* as a localized and burning pain and minimal bleeding in the elbow of the arm that rapidly disappeared. A few days after the bite, the victim presented labyrinthine syndrome, including rotator dizziness, nausea, and vomiting. Probably, these effects were related with the snakebite [70]. In addition, de Medeiros et al. [17] reported mild dizziness as a unique systemic manifestation found among 297 studied cases of bite by *P. patagoniensis*. For Chile, we found the case of a 25 year old man that was bitten in the finger by *P. chamissonis* and had intense pain, edema, vertigo, headache, fever, and debility [36]. Although dizziness or headache are considered a systemic effect, it may also be manifested in patients with anxiety or hyperventilation. 

On the other hand, the presence of lymphadenopathy in the affected limb suggests that venom absorption can occur through the lymphatic system, but blood levels of the venom should be minimal to pose a danger to the victim [71]. Frequently, venom is not inoculated by *Philodryas* species, because the opistogliph condition gives restricted access to the bite site, an event known as dry bite [72]. In Chile, we did not find formal reports concerning dry bites by *Philodryas* species.

### 2.5. Misidentification of Species and Wrong Diagnosis

Identification of offending species and objective description of symptoms are incomplete or confusing in most of the Chilean analyzed literature. Chilean clinical reports generally do not identify the offending species, leaving unclear whether it was involved a *Philodryas* or *Tachymenis* specimens (e.g., [73]). For an unidentified snake species involved in a clinical case described by Rayo [73], the specimen was later assigned to *Tachymenis peruviana* based on its total length (300 mm) by Neira et al. [31]; however, it is currently recognized that the size of Chilean snakes is not a correct criterion for taxonomic identification [48]. 

### 2.6. Consequences Due to the Snakebite by Philodryas Species: Sequels and Secondary Infections

Reports on the medical consequences or complications of bites by rear-fanged snakes are poor. Favorable sintomatological evolution in *Philodryas* species have been described without important consequences for the health of the victim, producing mild, such as for *P. viridissima* [18], to moderately severe effects such as for *P. olfersii* and *P. patagoniensis* [14,17]. For some snakebite cases produced by these two latter species, hands and arms remained with edema, pain, and partial loss of their movements for a prolonged time (15 days) and then recovered their normal appearance [13]. For a snakebite case by *P. chamissonis*, 10 days after, a discrete increase in the volume of the hand and residual ecchymosis in the arm were observed [37]. We did not find evidence of organic or functional consequences, including allergic reactions, produced for the *P. chamisonis* envenoming.

In certain cases, the snakebites produced abscesses containing aerobic and anaerobic bacteria and its multiplication can be favored by venom-inducing edema [74,75]. For *Philodryas* species, secondary infections were confirmed for three of 297 bite cases (1.0%) produced by *P. patagoniensis* [17] and one case reported about the edema produced by *P. olfersii* bite progressed to a wound with exudates which was treated with an anti-tetanus vaccine and daily medical control to prevent possible gangrene [13]. Although no infectious complications have been described [31], 43% of Chilean cases were correctly assigned to *P. chamissonis* snakebites and were prophylactically treated with antibiotics (e.g., cloxaciline, gentamicine, and penicillin) [36,37]. 

## 3. Conclusions 

Over 104 years, descriptions of Chilean *Philodryas* snakes have been published without analyzing venom properties, and mention of snake accidents only began to occur in 1938 [73,76] and did not include further details on the species. Our analysis of 185 years of literature on Chilean *Philodryas* species revealed that studies regarding taxonomy, ecology, and natural history represent 90% of the literature, positioning snakebites and venom toxins as neglected issues in Chile. 

Within the trans-Andean group of *Philodryas* genus, *P. chamissonis* is the only species widely distributed in high population density areas, such as the central-southern zone of Chile, inhabiting sites where the species may be in contact with human activity, increasing the possibility of snakebites as revealed by our data analysis of 185 locations (Figure 2). The reduced number of snakebite cases recorded in 185 years of literature (equivalent to one report of ophidic accident every 20.5 years) was correlated with a low incidence of snakebite accidents in Chile [31]; however, it may also be a consequence of under-reporting of cases due to the inadequate publication of ophidic accidents or scarce research in this area. Consistent with this idea, it is surprising that all reported cases of ophidic accident by *P. chamissonis* occurred in the Valparaíso and Metropolitan regions which represent a small portion of the distributional range of this species. A greater effort is required in the other areas of Chile to study this species. 

Several aspects of ophidism by Chilean species of the genus *Philodryas* remain unknown. The toxicological effects of venom and the clinical implications of a potential bite in humans by *P. simonsii* and *P. tachymenoides* are entirely unknown. Moreover, although different pharmacological options have been used successfully for treatment of snakebites by *P. chamissonis*, currently there is no official scheme despite proposed recommendations [31]. All ophidian accidents in Chile occurred in young people, mostly men, and the effects of envenoming and its clinical management in infants, pregnant women, and the elderly are currently unknown. It is also unknown if its presence in patients with non-communicable disease which are of high prevalence in Chile, such as cardiovascular diseases, could contribute to ophidism progression and its treatment. Finally, this review highlights the need to develop deep research in the toxicological aspects of snakebites by *Philodryas* species and urgent improvements to the management of this neglected disease in Chile.

## 4. Materials and Methods 

### 4.1. Recollection of Literature

A search of published articles between 1834 and 2019 on Web of Science, Scopus, and Google Scholar for the three species of the genus *Philodryas* in Chile was conducted using the keywords Chile* AND reptiles OR snakes OR serpientes OR culebra OR ophidism OR snakebite and the scientific names of the studied species (*P. chamissonis, P. simonsii,* and *P. tachymenoides*). All works published in peer-reviewed journals were included, and unpublished theses, seminars, and books were excluded. Works not available on digital platforms were obtained in the libraries of the University of Buenos Aires, Faculty of Veterinary Sciences (Buenos Aires, Argentina); Academia Colombiana de Ciencias Exactas, Físicas y Naturales (Bogotá, Colombia), Library of the Faculty of Medicine; University of Chile (Santiago, Chile), Library of Lucas Sierra Foundation (Viña del Mar, Chile); Library of the Medical Society of Santiago (Santiago, Chile), Michigan State University Archives and Historical Collections (East Lansing, MI, USA).

### 4.2. Data of Geographic Distribution and Location of Snakebite Cases

The locations of *P. chamissonis* were obtained from the literature [8,77,78,79,80,81,82,83,84,85,86,87,88,89,90,91,92,93,94,95,96,97,98,99] and examination of specimens from herpetological collections: Museo Nacional de Historia Natural (MNHN; Santiago, Chile) and the Museo Regional de Concepción (MRC; Concepción, Chile). The locations of snakebites were obtained from the literature. Maps were done using ARCGIS 10.7 software. Population density estimations (inhabitants per km^2^) were conducted using 2017 census results obtained from the Instituto Nacional de Estadísticas, Chile (INE). 

## Figures and Tables

**Table 1:** List of publications that describe clinical and toxicological aspects of venom of *Philodryas* species in Chile.

Species	Snakebite Cases	Toxicological Aspect and Description of Toxins
*P. chamissonis*	[31,35,36,37,38,42,57]	[29,43,44]
*P. simonsii*	No reported	No reported
*P. tachymenoides*	No reported	No reported

## Supplementary Materials

The following are available online at https://www.mdpi.com/2072-6651/11/12/697/s1, Table S1: Examined specimens, Table S2: list of literature analyzed.
