# Supplementary material for: Philodryas (Serpentes: Dipsadidae) Envenomation, A Neglected Issue in Chile
Source: Toxins (Basel). 2019 Nov 29;11(12):697. doi: 10.3390/toxins11120697 (PMC6950111; doi:10.3390/toxins11120697)
Supplement: Supplementary file 1 [file toxins-11-00697-s001.pdf]

Supplementary Materials: Philodryas (Serpentes: Dipsadidae) envenomation, a neglected issue in Chile.

Félix A. Urra, Alejandro Bruno Miranda-Calle and Ramiro Araya-Maturana

Table S1: Examined Specimens. *Philodryas chamissonis* - (Wiegmann, 1835) –.**COLECCIÓN MUSEO NACIONAL DE HISTORIA NATURAL DE CHILE**

(MNHN-Chile)

MNHNCL HERP 0133. País: Chile, Región: Valparaíso, Provincia: Valparaíso, Comuna: Quintero, Localidad: Bosque Las Petras. Recol.: Núñez, H., 25/may/1979.

MNHNCL HERP 0148-0149. País: Chile, Región: Metropolitana de Santiago,Provincia: Santiago, Comuna: Peñalolén, Localidad: San Luis de Macul. Recol.: Núñez, H., 3/nov/1979.

MNHNCL HERP 0489. País: Chile, Región: Metropolitana de Santiago, Provincia: Chacabuco, Comuna: Colina, Localidad: Colina. Recol.: Vizcarra, O., 8/oct/1978.

MNHNCL HERP 0490. País: Chile, Región: Metropolitana de Santiago,Provincia: Maipo, Comuna: San Bernardo, Localidad: Cerro Chena. Recol.: Arancibia, L., 22/jul/1976.

MNHNCL HERP 0491. País: Chile, Región: Metropolitana de Santiago, Provincia: Santiago, Comuna: Peñalolén, Localidad: Quebrada de Macul, parte alta. Recol.: Godoy, M., 24/nov/1976.

MNHNCL HERP 0492. País: Chile, Región: Metropolitana de Santiago, Provincia: Chacabuco, Comuna: Lampa, Localidad:Taco de Lampa. Recol.: Núñez, H., 14/sep/1980

MNHNCL HERP 0493. País: Chile, Región: Metropolitana de Santiago,Provincia: Cordillera, Comuna: Pirque, Localidad: Santa Rita. Recol.: Núñez, H. & Jarpa, M., 15/ago/1975.

MNHNCL HERP 0496. País: Chile, Región: Metropolitana de Santiago, Provincia: Maipo, Comuna: San Bernardo, Localidad: Cerro Chena. Recol.: Contreras, L., 5/sep/1976

MNHNCL HERP 0497. País: Chile, Región: Metropolitana de Santiago, Provincia: Santiago, Comuna: Peñalolén, Localidad: San Luis de Macul. Recol.: Núñez, H., 8/feb/1977.

MNHNCL HERP 0500. País: Chile, Región: Metropolitana de Santiago,Provincia: Cordillera, Comuna: Pirque, Localidad:Lo Arcaya. Recol.: Aspillaga, E. & Rodríguez, G. & Zafira, Y.,25/abr/1977.

MNHNCL HERP 0501. País: Chile, Región: Metropolitana de Santiago,Provincia: Santiago, Comuna: La Reina, Localidad:Cerros de la La Reina. Recol.: Núñez, H., 3/abr/1976

MNHNCL HERP 0503-0505. País: Chile, Región: Valparaíso, Provincia: Valparaíso, Comuna: Concón, Localidad: Concón. Recol.: Núñez, H., 29/nov/1980.

MNHNCL HERP 0553. País: Chile, Región: Metropolitana de Santiago,Provincia: Melipilla, Comuna: Melipilla, Localidad: Bollenar. Recol.: Sallaberry, M., 7/dic/1980.

MNHNCL HERP 0554. País: Chile, Región: Coquimbo, Provincia: Choapa,Comuna: Los Vilos, Localidad: Frente a Isla de Lobos. Recol.: Gajardo, C., 2/ene/1981.

MNHNCL HERP 0578. País: Chile, Región: Coquimbo, Provincia: Choapa, Comuna: Los Vilos, Localidad: Frente a Isla de Lobos. Recol.: Gajardo, M., 2/feb/1981.

MNHNCL HERP 0984. País: Chile, Región: Del Biobío, Provincia: Ñuble, Comuna: Cobquecura, Localidad: Cobquecura. Recol.: Yáñez, J. & Gajardo, M., 20/mar/1981.

MNHNCL HERP 0985. País: Chile, Región: Metropolitana de Santiago, Provincia: Santiago, Comuna: Lo Barnechea, Localidad: Lo Barnechea. Recol.: Berner, C., 6/jun/1981.

MNHNCL HERP 0986. País: Chile, Región: Coquimbo, Provincia: Choapa,Comuna: Los Vilos, Localidad: Frente a Isla de Lobos. Recol.: Arriagada, G., 24/jul/1981.

MNHNCL HERP 1037-1038. País: Chile, Región: Coquimbo, Provincia: Limarí, Comuna: Ovalle, Localidad: Fray Jorge, Parque Nacional. Recol.: Yáñez, J., 30/oct/1981.

MNHNCL HERP 1039. País: Chile, Región: Coquimbo, Provincia: Limarí,Comuna: Ovalle, Localidad: Km 300, Carretera Panamericana Norte. Recol.: Yáñez, J., 30/oct/1981

MNHNCL HERP 1068. País: Chile, Región: Metropolitana de Santiago, Provincia: Santiago, Comuna: Peñalolén, Localidad: Peñalolén. Recol.: Núñez, H., 26/dic/1981.

MNHNCL HERP 1069. País: Chile, Región: Metropolitana de Santiago,Provincia: Santiago, Comuna: Cerrillos, Localidad: Ex escuela Arquitectura. Recol.: Salinas, S., 16/nov/1981.

MNHNCL HERP 1112. País: Chile, Región: Valparaíso, Provincia: Valparaíso, Comuna: Concón, Localidad: Concón. Recol.: Núñez, H. & Yáñez, J., 25/mar/1982.

MNHNCL HERP 1211. País: Chile, Región: Metropolitana de Santiago,Provincia: Santiago, Comuna: Vitacura, Localidad: Cerro Manquehue. Recol.: Núñez, H. & Labra, J. & Labra, A., 5/sep/1982.

MNHNCL HERP 1212. Det.: Núñez, H., 29/oct/1982. País: Chile, Región: Coquimbo, Provincia: Choapa, Comuna: Los Vilos, Localidad: Quebrada El Roble. Recol.: Yáñez, J., 10/oct/1982.

MNHNCL HERP 1213-1215. País: Chile, Región: Del Maule, Provincia: Talca,Comuna: Talca, Localidad: Camarico. Recol.: Núñez, H. & Labra, J. & Labra, A., 23/oct/1982.

MNHNCL HERP 1392. País: Chile, Región: Coquimbo, Provincia: Choapa, Comuna: Illapel, Localidad: Cuesta Cavilolén. Recol.: Fuentes, E., 1/ene/1977. Obs.: Ex Colección E. Fuentes.

MNHNCL HERP 1597. País: Chile, Región: Metropolitana de Santiago, Provincia: Santiago, Comuna: Macul, Localidad: Calle Joaquín Rodríguez. Recol.: ND, 31/jul/1983.

MNHNCL HERP 1698. País: Chile, Región: Metropolitana de Santiago, Provincia: Santiago, Comuna: Pudahuel, Localidad: Pudahuel. Recol.: Caro, V., 13/ene/1900.

MNHNCL HERP 1700. País: Chile, Región: Valparaíso, Provincia: Valparaíso, Comuna: Concón, Localidad: Concón. Recol.: Yáñez, J., 25/oct/1980.

MNHNCL HERP 1782. País: Chile, Región: Del Libertador General Bernardo O'Higgins, Provincia: Cachapoal, Comuna: San Vicente, Localidad: San Vicente de Taguatagua. Recol.: Cabezas, J., 23/feb/1986.

MNHNCL HERP 1793-1794. País: Chile, Región: Del Maule, Provincia: Talca, Comuna: San Clemente, Localidad: 4 km al Este de la Mina Talca. Recol.: Contreras, L., 23/nov/1984.

MNHNCL HERP 1895. País: Chile, Región: Metropolitana de Santiago, Provincia: Santiago, Comuna: Santiago, Localidad: Santiago. Recol.: Codoceo R., M., 31/mar/1955.

MNHNCL HERP 1999. País: Chile, Región: Del Libertador General Bernardo O'Higgins, Provincia: Cachapoal, Comuna: Las Cabras, Localidad: Lago Rapel, Sector El Estero. Recol.: Braemer V., A., 25/feb/1990.

MNHNCL HERP 2001. País: Chile, Región: Del Libertador General Bernardo O'Higgins, Provincia: Colchagua, Comuna: San Fernando, Localidad: Termas del Flaco. Recol.: Labra, A. & Núñez, H., 18/feb/1990.

MNHNCL HERP 2003. País: Chile, Región: Del Libertador General Bernardo O'Higgins, Provincia: Colchagua, Comuna: San Fernando, Localidad: Termas del Flaco. Recol.: González, E., 17/feb/1990.

MNHNCL HERP 2160. País: Chile, Región: Metropolitana de Santiago, Provincia: Cordillera, Comuna: San José de Maipo, Localidad: Cajón del Maipo. Recol.: ND, 31/ene/1991.

MNHNCL HERP 2291. País: Chile, Región: Antofagasta, Provincia: Antofagasta, Comuna: Taltal, Localidad: Rinconada de Paposo. Recol.: Gálvez, O. & Cornejo, A. & Villarroel, M.,1/dic/1989.

MNHNCL HERP 2498. País: Chile, Región: Valparaíso, Provincia: San Antonio, Comuna: El Quisco, Localidad: El Quisco. Recol.: Codoceo R., M., 2/ene/1955.

MNHNCL HERP 2583. País: Chile, Región: Metropolitana de Santiago, Provincia: Santiago, Comuna: Santiago, Localidad: Santiago. Recol.: ND, 30/abr/1993.

MNHNCL HERP 2989. País: Chile, Región: Coquimbo, Provincia: Choapa, Comuna: Salamanca, Localidad: Mina Los Pelambres. Recol.: Núñez, H. & Yáñez, J., 4-8/ago/1997.

MNHNCL HERP 3543. Det.: Núñez, H., ago/2002. País: Chile, Región: Metropolitana de Santiago, Provincia: Cordillera, Comuna: Pirque, Localidad: Pirque. Recol.: Niemeyer, H., 31/dic/2001

MNHNCL HERP 3544. Det.: Núñez, H., ago/2002. País: Chile, Región: Metropolitana de Santiago, Provincia: Cordillera, Comuna: Puente Alto, Localidad: Las Vizcachas. Recol.: Labra, A. & Escobar, C., 31/dic/1999.

MNHNCL HERP 3545. Det.: Núñez, H., ago/2002. País: Chile, Región: Valparaíso, Provincia: Marga Marga, Comuna: Olmué, Localidad: Ocoa. Recol.: Niemeyer, H., 31/dic/2000.

MNHNCL HERP 3807. País: Chile, Región: Coquimbo, Provincia: Limarí, Comuna: Ovalle, Localidad: Fray Jorge, Parque Nacional. Recol.: Rojas, G., 26/oct/2002.

MNHNCL HERP 4335. País: Chile, Región: Metropolitana de Santiago, Provincia: Santiago, Comuna: Renca, Localidad: Río Mapocho. Recol.: ND, 22/may/1980. Obs.: Ex Colección DBGUCH.

MNHNCL HERP 4346. País: Chile, Región: Metropolitana de Santiago, Provincia: Santiago, Comuna: Peñalolén, Localidad: Peñalolén. Recol.: ND, 15/oct/1985. Obs.: Ex Colección DBGUCH

MNHNCL HERP 4347. País: Chile, Región: Metropolitana de Santiago, Provincia: Santiago, Comuna: Pudahuel, Localidad: Pudahuel. Recol.: ND, 16/jun/1985. Obs.: Ex Colección DBGUCH.

MNHNCL HERP 4408. Det.: Veloso, A., 7/dic/1981. País: Chile, Región: Metropolitana de Santiago, Provincia: Santiago, Comuna: Lo Barnechea, Localidad: La Dehesa. Recol.: Veloso, A., 7/dic/1981. Obs.: Ex Colección DBGUCH.

MNHNCL HERP 4409. Det.: Veloso, A., 30/jun/1982. País: Chile, Región: Metropolitana de Santiago, Provincia: Santiago, Comuna: Recoleta, Localidad: Cerro San Cristóbal. Recol.: ND, 30/jun/1982. Obs.: Ex Colección DBGUCH.

MNHNCL HERP 4410. Det.: Veloso, A., 17/sep/1981. País: Chile, Región: Coquimbo, Provincia: Limarí, Comuna: Monte Patria, Localidad: El Palqui. Recol.: Egaña, P. & Moreno, E., 17/sep/1981. Obs.: Ex Colección DBGUCH.

MNHNCL HERP 4411. Det.: Veloso, A., 15/nov/1981. País: Chile, Región: Metropolitana de Santiago, Provincia: Cordillera, Comuna: Pirque, Localidad: La Puntilla. Recol.: Moreno, R., 15/nov/1981. Obs.: Ex Colección DBGUCH.

MNHNCL HERP 4413. Det.: Navarro, J., 18/feb/1982. País: Chile, Región: Valparaíso, Provincia: Valparaíso, Comuna: Puchuncaví, Localidad: Maitencillo. Recol.: Navarro, J. & Moreno, R., 18/feb/1982. Obs.: Ex Colección DBGUCH.

MNHNCL HERP 4414. Det.: Veloso, A., 26/abr/1981. País: Chile, Región: Metropolitana de Santiago, Provincia: Santiago, Comuna: Estación Central, Localidad: Carlos Valdovinos, Sector Buzeta. Recol.: Veloso, A., 26/abr/1981. Obs.: Ex Colección DBGUCH.

MNHNCL HERP 4494. Det.: Núñez, H., 30/jun/2010. País: Chile, Región: Metropolitana de Santiago, Provincia: Santiago, Comuna: Recoleta, Localidad: Cerro San Cristóbal, ladera Norponiente, Recol.: Sánchez, M., 18/sep/1981.

MNHNCL HERP 5024-5029. Det.: ND, 1984. País: Chile, Región: ND, Provincia: ND, Comuna: ND, Localidad: Chile Central. Recol.: ND, 1984. Obs.: Ex Colección DBGUCH.

MNHNCL HERP 5030. Det.: ND, 1981. País: Chile, Región: Coquimbo, Provincia: Limarí, Comuna: Ovalle, Localidad: Huanillo. Recol.: Escobar, F., 18/sep/1981. Obs.: Ex Colección DBGUCH.

Philodryas chamissonis **- (Wiegmann, 1835) –**

Museo Regional de Concepción (MRC)

MRC 0258 Cerro Negro. Quillón. 10-11-1981.

MRC 0260 Isla Mancera. Valdivia. 03-02-1973.

MRC 0261 Huasco Bajo, Huasco. 09-05-1983.

MRC 0263 Coelemu. 19-03-1979.

MRC 0264 Coelemu. 19-03-1979.

MRC 0265 Paso Hondo. Cabrero. 11-04-1982.

MRC 0266 Huillinco Norte. Cañete. 17-02-1977.

MRC 0267 Lomas Coloradas. Coronel. 18-02-1982.

MRC 0277 Laraquete. 15-02-1984.

Table S2: list of literature analyzed.

Article	Year	Authors	Full reference
**1**	1834	Wiegmann, A. F. A	In: Dr. F. J. F. Meyen: Beiträge zur Zoologie gesammelt auf einer Reise um die Erde. Siebente Abhandlung. Amphibien. Nova Acta Physico-Medica Academia Caesarea Leopoldino-Carolina (Halle) 17: 185-268 [1835]
**2**	1837	Schlegel, H.	Essai sur la physionomie des serpens. Partie Descriptive. La Haye (J. Kips, J. HZ. et W. P. van Stockum), 606 S. + xvi
**3**	1855	Girard, C.	Abstract of a report to Lieut. James M. Gilliss, U.S.N., upon the reptiles collected during the U.S.N. Astronomical Expedition to Chili. Proc. Acad. Nat. Sci. Philadelphia 7 [1854]: 226-227
**4**	1860	Philippi, Rodulfo Amando	Viage al Desierto de Atacama Reptiles 148-151. 1 Lámina
**5**	1860	Philippi, Rodulfo Amando	Reise durch die Wüste Atacama auf Befehl der chilenischen Regierung im Sommer 1853-54. Unternommen und Beschrieben von Rudolph Amandus Philippi. Halle: Eduard Anton
**6**	1864	Philippi, Rodulfo Amando	Descripción de tres especies neuvas de reptiles Chilenos. Anales Univ. Chile 8 (1): 744-748
**7**	1894	Boulenger, George A.	Catalogue of the snakes in the British Museum (Natural History). Volume II., Containing the Conclusion of the Colubridæ Aglyphæ. British Mus. (Nat. Hist.), London, xi, 382 pp.
**8**	1899	Philippi, R.A.	Serpientes de Chile & Descripciones breves de dos especies nuevas de sapo (Bufo). Anales Universidad de Chile 104: 723-725
**9**	1899	Philippi, Rodulfo Amando	Sobre las serpientes de Chile. Anales Univ. Chile 104: 715-723
**10**	1900	Boulenger, George A.	Descriptions of new batrachians and Reptiles collected by Mr. P. O. Simons in Peru. Ann. Mag. Nat. Hist. (7) 6 (32): 181-186
**11**	1916	Quijada, Bernardino	Catálogo sistemático de los reptiles chilenos y extranjeros conservador en el Museo de Historia Natual. Boletín Museo Nacional 22-47
**12**	1932	Parker, H.W.	Some new or rare reptiles and amphibians from southern Ecuador. Ann. Mag. Nat. Hist.1932, (10) 9: 21-26
**13**	1937	Helmich,W.	Anotaciones para el conocimiento de las culebras de chile. Rev. Chil. Hist. Nat. 41, 1937. 107-110.
**14**	1938	Johow A.	Mordedura de culebra. Bol Soc Cirugía (Rev Méd Chile) 1938; 66: 661-3.
**15**	1938	Rayo F, Covarrubias R, Ruiz M.	Mordedura de serpiente. Bol Soc Cirugía (Rev Méd Chile) 1938; 66: 773-9.
**16**	1940	Gigoux E R	Los ofidios chilenos. Santiago de Chile. Bol Museo Nacional Hist Natural 1940; 18: 5.
**17**	1943	Schmidt,KP., Walker,WF.	Snakes of the Peruvian coastal region. Zool. Ser. Field Mus. nat. Hist., Chicago, 1943, 24: 297-327
**18**	1943	Schmidt, K. P.; W. F. Walker, Jr.	Three new snakes from the Peruvian Andes. Field Museum of Natural History, Chicago - Zoological Series 24 (28): 325-329
**19**	1943	Schmidt, Karl P. & Walker, Warren F.	Peruvian snakes from the University of Arequipa. Zoological Series of Field Museum of Zoology 24 (26): 279-296

**20**	1944	Luer, HG.	Un caso teratológico en un ofidio chileno. Bol. Soc. Biol. Concep. 19, 1944. 83-86.
**21**	1945	Pflaumer, K.	Un segundo hallazgo de una culebra con dos cabezas en Chile. Rev. Chil. His. Nat.48, 1945. 97-102
**22**	1947	Gajardo- Tobar R	¿Los ofidios chilenos son capaces de envenenar?. Bol Hosp Viña del Mar (Chile) 1947; 3: 43-51.
**23**	1950	Donoso-Barros, R y Candiani, S.	Reptiles de la provincia de santiago. Rev. Acad. Colomb. Ciencias Exac. 1950. 7, 28, 482-489.
**24**	1950	Codoceo, M.	Reptiles de Tarapaca. (Nota preliminar). Invest. Zool. Chilenas 1: 15.
**25**	1954	Schenone H, Bertín V, Mann G.	Un nuevo caso de ofidismo. Bol Chil Parasitol 1954; 9:88-9.
**26**	1958	Codoceo, M.	Distribución geográfica de las serpientes ponzoñozas. Not. Mens. Mus. Hist. Nat.II, 1958. 12, 5-7
**27**	1958	Gajardo- Tobar R	Cinco casos de ofidismo. Bol Hosp Viña del Mar (Chile) 1958; 15: 25-38.
**28**	1958	Marx,H	Catalogue of type specimens of reptiles and amphibians in Chicago Natural History Museum. Fieldiana Zool. 36: 407-496
**29**	1959	Donoso-Barros, R, Cardenas,S.	Estudio del veneno de dromicus chamissonis (wiegmann), inv, zool. chil. 1959. 5, 93-95.
**30**	1960	Peters , J. A.	The snakes of Ecuador; check list and key. Bull. Mus. Comp. Zool. Harvard, 1960, 122: 489-541
**31**	1961	Donoso-Barros, R.	The Reptiles of the Lund University Chile Expedition. Copeia 4, 486-488.
**32**	1961	Donoso-Barros, R.	Emponzoñamiento por ofidios chilenos. Rev Pediatr Clin Soc 1961; 1: 65-79.
**33**	1962	Donoso-Barros, R.	Los ofidios chilenos. Not. Mens. Mus. Hist. Nat. 1962; 6. 66, 3-8
**34**	1962	Donoso-Barros, R, Cardenas,S.	El veneno de las culebras chilenas. Not. Mens. Mus. Hist. Nat, 1962. 74, 2-4.
**35**	1964	Donoso-Barros, R.	Ecologia de los reptiles del sur de Coquimbo. Zooiatría 3: 11, 2-4
**36**	1965	Donoso-Barros,R., Cardenas,S.	Los tipos de serpientes de Rodulfo Armando Philippi, en el Museo De Historia Natural de Santiago de Chile. Actas Ii Congr. Sudam. Zool.1965. 2, 225-234.
**37**	1965	Schenone H, Reyes H.	Animales ponzoñosos de Chile. Bol Chil Parasitol 1965; 20: 104-9
**38**	1965	Donoso-Barros, R. y Sergio Cárdenas	Los tipos de serpiente de Rodulfo Amando Philippi, en el Museo de Historia Natural de Santiago de Chile. Actas 2º Conreso Latinoam. Zoologia. 2; 225-234. Sao Paulo. Brasil
**39**	1969	Webb, RG., Greer, JK.	Amphibians and reptiles from Malleco Province, Chile. Publ. Mus. Michigan State Univ. Biol. Ser. 1969, 4: 193-226.
**40**	1969	Dowling H.G.	The hemipenis of Philodryas Günther: a correction (Serpentes, Colubridae). American Museum Novitates 2375: 1-6.
**41**	1970	Donoso-Barros, R.	Catálogo herpetológico chileno. Boletín del Museo Nacional de Historia Natural, Chile 31: 49-124 (1970)
**42**	1974	Donoso-Barros, R.	Nuevos reptiles y anfibios de Chile. Bol. Soc. Biol. Concepción (Chile), 1974, 48: 217-229.
**43**	1977	Thomas, R.A.	A New Generic Arrangement for Incaspis and Mainland South American Alsophis and the Status of Two Additional Peruvian Species. Copeia 1977 (4): 648-652

**44**	1980	Dixon, J. R.	The neotropical colubrid snake genus Liophis. The generic concept. Milwaukee Public Museum Contributions in Biology and Geology 31: 1-40
**45**	1980	Yáñez, J., H. Núñez, R. P. Schlatter & F. M. Jaksic.	Prey of the Harris´ Hawk in Central Chile. Auk (1980) 97: 629–631.
**46**	1981	Jaksié, F.M., Greene, H.W., Yáñez, J.L.	The guild structure of a community of predatory vertebrates in central Chile (1981) Oecologia, 49 (1), pp. 21-28. DOI: 10.1007/BF00376893
**47**	1981	Jaksic, F., Nuñez, H., Yañez	Nuevo cambio taxonómico para la culebra de cola larga (Reptilia, Ophidia, Colubridae). Not. Mens. Mus.Nac. Hist. Nat. (Chile),1981, 293: 12.
**48**	1982	Simonetti, J., H. Núñez & J. Yañez	Falco sparverius L.: Rapaz generalista en Chile central. Boletín del Museo Nacional de Historia Natural (1982) 39: 119–124.
**49**	1984	Nuñez, H	Culebras chilenas: más allá del mito. Revista Naturaleza (Chile)1984, 2 (9): 16-19.
**50**	1987	Troncoso, JF., Ortiz, JC.	Catálogo Herpetológico del Museo Regional de Concepción. Comunicaciones del Museo Regional de Concepción (Chile), 1987, 1: 9-19.
**51**	1987	Moreno R, Navarro J, Iturra P, Veloso AM.	The karyotype of Philodryas chamissonis (Colubridae). Identification of nucleolar organizer region (NOR) and sex chromosomes by banding methods. Braz. J Genetics,1987, 3: 497-506.
**52**	1987	Jaksic FM & M Delibes	A comparative analysis of food – niche relationships and trophic guild structure in two assemblages of vertebrate predators differing in species richness: causes, correlations and consequences. Oecologia 71:461-472
**53**	1988	Medel, R.G., Jimenez, J.E., Fox, S.F., Jaksic, F.M.	Experimental evidence that high population frequencies of lizard tail autotomy indicate inefficient predation (1988) Oikos, 53 (3), pp. 321-324.
**54**	1988	Bozinovic, F., Rosenmann, M.	Energetics and food requirements of the female snake Phillodryas chamissonisduring the breeding season. Oecologia, 1988, 75: 282-284.
**55**	1988	Veloso, A., Navarro, J.	Lista sistemática y distribución geográfica de anfibios y reptiles de Chile. Bull. Mus. Reg. Sei. Nat. Torino, 1988, 6(2): 481-539.
**56**	1988	Ortíz, JC.	Situación de la exportación de los vertebrados terrestres chilenos. Comunicaciones del Museo Regional de Concepción (Chile), 1988, 2: 37-41.
**57**	1990	Jimenez J, Jaksic F.	Historia Natural del Aguila Geranoaetus melanoleucus: una revisión. El Hoernero(Buenos Aires) - Revista de Ornitología Neitropical. (1990) 13, 2: 97-110,
**58**	1990	Medel, R. G., P.A. Marquet, S. F. Fox. & F. M. Jaksic.	Depredación sobre lagartijas en Chile central: importancia relative de atributos ecológicos y morfológicos Revista Chilena de Historia Natural 63: 261-266
**59**	1991	Lazo, I., Anabalón, J.	Nesting of the common Diuca finch in the central Chilean scrub. Wilson Bulletin, 1991, 103: 143-146.
**60**	1992	Greene, HW., Jaksic, FM.	The feeding behavior and natural history of two Chilean snakes, Philodryas chamissonis and Tachymenis chilensis (Colubridae). Revista Chilena de Historia Natural, 1992, 65: 485-493.
**61**	1992	Nuñez, H	Geographical data of Chilean lizards and snakes in the Museo Nacional de Historia Natural, Santiago, Chile.

			Smithsonian Herpetological Information Service, 1992, 91: 1-29.
**62**	1992	Habit, EM., Ortiz, JC., Victoriano, P.	Osteología craneana de Philodryas chamissonis (Wiegmann, 1834) (Colubridae, Serpentes). Boletín de la Sociedad de Biología de Concepción (Chile), 1992, 63: 83-92.
**63**	1992	Nuñez, H., Jaksic, F.	Lista comentada de los reptiles terrestres de Chile Continental. Bol. Mus. Nac. Hist. Nat. Chile, 43: 63-91 (1992)
**64**	1993	Jaksic FM, P Fisinger y JE Jiménez	A long term study on the dyamics of guild structure among predatory vertebrates at a semi-arid Neotropical site Oikos 67: 87-96
**65**	1994	Lobo, F., Scrocchi, G.	Osteología craneal del género Philodryas (Serpentes: colubridae). Cuadernos de Herpetología; 1994, 8, 1, 104-111.
**66**	1994	Arzola J, Schenone H.	Dos casos nuevos de ofidismo en Chile. Bol Chil Parasitol 1994; 49: 69-70.
**67**	1995	Carrillo De Espinoza, N., Icochea, J.	Lista taxonómica preliminar de los reptiles vivientes del Perú. Publ. Mus. Hist. Nat. UNMSM, 1995, 49:1-27
**68**	1996	Díaz, I & J. A. Simonetti.	Vertebrados en áreas silvestres protegidas; reptiles de la Reserva Nacional Río Clarillo, Chile Central. Vida Silvestre Neotropical 5 2: 140-142
**69**	1997	Thomas, R.A.	Galapagos Terrestrial Snakes: Biogeography and Systematics. Herp. Nat. Hist. 5(1): 19-40. 1997.
**70**	1999	Kuch, U.	Notes on two cases of human envenomation by the South American colubrid snakes Philodryas olfersi latirostris COPE, 1862 and Philodryas chamissonis (WIEGMANN, 1834). Herpetozoa, 1999, 12 (1-2): 11-16
**71**	2000	Moreno, R., Moreno, J., Torres-Pérez, F. & J.C. Ortiz.	Reptiles del Parque Nacional “Nevados de Tres Cruces” (III Región, Chile). Boletín Sociedad Biología de Concepción (Chile) 71: 41–43.
**72**	2001	Grehan, John	Biogeography and evolution of the Galapagos: integration of the biological and geological evidence. Biological Journal of the Linnean Society 74: 267–287
**73**	2001	Moreno M, Rodrigo, Moreno, Jorge, Torres-Pérez, Fernando, Ortiz, Juan C., & Breskovic, Antonio.	Catálogo Herpetológico del Museo del Mar de la Universidad Arturo Prat de Iquique, Chile. Gayana. 65 (2) 2001
**74**	2002	Moreno, R., Moreno, J., Ortiz, JC., Victoriano, P., Torres-Pérez, F.	Herpetofauna del Parque Nacional Llanos de Challe (III Región, Chile). Gayana, 2002, 66: 7-10.
**75**	2003	Pincheira-Donoso, D	Record of the colubrid snake Philodryas simonsii Boulenger from Chile (2003) Herpetological Bulletin, (84), p. 20
**76**	2003	Escobar, MA., Vukasonic, MA.	Depredación de Philodryas chamissonis (Serpentes:Colubridae) sobre polluelos de Aphrastura spinicauda (Passeriformes: Furnariidae): ¿Una culebra arborícola?. Not. Men. Mus. Hist. Nat. 2003, 352:18-20
**77**	2004	Labra, A., Niemeyer, H.M.	Variability in the assessment of snake predation risk by Liolaemus lizards (2004) Ethology, 110 (8), pp. 649-662. DOI: 10.1111/j.1439-0310.2004.01005.x
**78**	2005	Fredes, F., Raffo, E.	Raillitiella sp. finding on chilean long tailed snake (Philodryas chamissonis) from a zoo of the Metropolitan Region (2005) Parasitologia Latinoamericana, 60 (3-4), pp. 189-191.

**79**	2006	Sepulveda, M., Vidal, MA., Fariña, JM.	Microlophus atacamensis Predation. Herpetological Review, 2006, 37: 224–225.
**80**	2007	Mella, J.E.	Reptiles from "El Morado" natural monument (Region Metropolitana, Chile): Relative abundance, altitudinal distribution, and preference by different sizes of rocks (2007) Gayana, 71 (1), pp. 16-26.
**81**	2007	Aguilar, C., Lundberg, M., Siu-Ting, K., Jiménez, ME.	Nuevos registros para la herpetofauna del departamento de Lima, descripción del renacuajo de Telmatobius rimac Schmidt, 1954 (Anura: Ceratophrydae) y una clave de los anfibios. Rev. peru biol. v.14 n.2 Lima dic. 2007
**82**	2007	Neira, O.P., Jofre, M.L., Oschilewski, L.D., Subercaseaux, S.B., Munoz, S.N.	Mordedura por Philodryas chamissonis. Presentacion de un caso y revision de la literatura. Rev. Chil. Infectol. 2007, 24, 236-241.
**83**	2008	Zaher, H., Scrocchi, G., Masiero, R.	Rediscovery and redescription of the type of Philodryas laticeps Werner, 1900 and the taxonomic status of P. oligolepis Gomes, 1921 (Serpentes, Colubridae).Zootaxa 1940: 25–40 (2008).
**84**	2009	Lobos, G., Escobar, MAH., Thomson, RF., Alzamora, A.	Philodryas chamissonis (long-tailed snake) and Liolaemus nitidus. Predation determined by pit tag. Herpetol. Rev. 40: 358.
**85**	2011	Sallaberry-Pincheira, N., Garin, C.F., González-Acuña, D., Sallaberry, M.A., Vianna, J.A.	Genetic divergence of Chilean long-tailed snake (Philodryas chamissonis) across latitudes: Conservation threats for different lineages (2011) Diversity and Distributions, 17 (1), pp. 152-162. DOI: 10.1111/j.1472-4642.2010.00729.x
**86**	2011	Troncoso-Palacios, J. & Y. Marambio.	Lista comentada de los reptiles de la Región de Atacama. Boletín del Museo Regional de Atacama 2: 60–78
**87**	2012	Grazziotin, F.G., Zaher, H., Murphy, R.W., Scrocchi, G., Benavides, M.A., Zhang, Y.-P.,Bonatto, S.L.,	Molecular phylogeny of the New World Dipsadidae (Serpentes:Colubroidea): a reappraisal. Cladistics, 2012, 28, 437-459.
**88**	2013	Muñoz-Leal, S., Ardiles, K., Figueroa, R.A., González-Acuña, D.	Philodryas chamissonis (Reptilia: Squamata: Colubridae) preys on the arboreal marsupial Dromiciops gliroides (Mammalia: Microbiotheria: Microbiotheriidae) (2013) Brazilian Journal of Biology, 73 (1), pp. 15-17. DOI: 10.1590/S1519-69842013000100003
**89**	2013	Skewes, O., Acuña, L., San Martín-Órdenes, J.	Depredación de polluelos de chercán (Troglodytes aedon) por la culebra de cola larga (Philodryas chamissonis). Bole Chileno de Ornitología. Unión de Ornitólogos de Chile. 2013, 19 (1-2): 30-33.
**90**	2014	Zaher, H., Arredondo, J.C., Valencia, J.H., Arbeláez, E., Rodrigues, M.T., Altamirano-Benavides, M.	A new Andean species of Philodryas (Dipsadidae, Xenodontinae) from Ecuador (2014) Zootaxa, 3785 (3), pp. 469-480. DOI: 10.11646/zootaxa.3785.3.8
**91**	2014	Uribe, S., Estades, C.F.	Reptiles in monterey pine plantations of the Coastal Range of Central Chile (2014) Revista Chilena de Historia Natural, 87 (1), art. no. 25, .DOI: 10.1186/s40693-014-0025-z
**92**	2014	Troncoso-Palacios, J.	Nueva lista actualizada de los reptiles terrestres de la Región de Atacama, Chile. Boletín Chileno de Herpetología, 2014, 1: 1-4

**93**	2015	Urra, F.A., Pulgar, R., Gutiérrez, R., Hodar, C., Cambiazo, V., Labra, A.	Identification and molecular characterization of five putative toxins from the venom gland of the snake Philodryas chamissonis (Serpentes: Dipsadidae) (2015) Toxicon, 108, art. no. 5202, pp. 19-31. DOI: 10.1016/j.toxicon.2015.09.032
**94**	2015	Labra, A., Hoare, M	Chemical recognition in a snake–lizard predator–prey system (2015) Acta Ethologica, 18 (2), pp. 173-179.DOI: 10.1007/s10211-014-0203-7
**95**	2015	Venzal, J.M., González-Acuña, D., Muñoz-Leal, S., Mangold, A.J., Nava, S.	Two new species of Ornithodoros (Ixodida; Argasidae) from the Southern Cone of South America (2015) Experimental and Applied Acarology, 66 (1), pp. 127-139. DOI: 10.1007/s10493-015-9883-
**96**	2015	Jara, M., Pincheira-Donoso, D.	The neck flattening defensive behaviour in snakes: First record of hooding in the South American colubrid genus Philodryas (2015) Animal Biology, 65 (1), pp. 73-79. DOI: 10.1163/15707563-00002459
**97**	2015	Castro-Pastene, C., Carrasco, H., & Troncoso-Palacios, J.	Lagartijas y serpientes del Parque Nacional Radal Siete Tazas. Boletín Chileno de Herpetología. 2: 12-16.
**98**	2016	Ruiz de Gamboa, M	Lista actualizada de los reptiles de Chile. Boletín Chileno de Herpetología. 3: 7-12.
**99**	2017	Torres, F	Observación de culebra de cola larga, Philodryas chamissonis (Wiegmann 1835) (Squamata: Dipsadidae) depredando sobre un polluelo de yal (Phrygilus fruticeti). Boletín Chileno de Herpetología. 4: 21.
**100**	2017	Reyes-Olivares, R; Sepulveda-Luna, E., Labra,A.	Philodryas chamissonis (Chilean Green Racer). Diet. Herpetological review 48 (4) 2017: 865-866.
**101**	2018	Constanzo-Chávez, J., Penna, M., Labra, A.	Comparing the antipredator behaviour of two sympatric, but not syntopic, Liolaemus lizards. Behav Processes. 2018;148:34-40. doi: 10.1016/j.beproc.2018.01.005.
**102**	2018	San Martín-Ordenes, J., Gonz+alez-Acuña, D.	Zoocide of a Chilean green racer Philodryas chamissonis (Serpentes: Dipsadinae) for preying nestlings of house wren (Troglodytes aedon: Aves): Biophilia with double standard?. GestiónAmbiental, 2018, 35: 5-11.
**103**	2018	Blanco, JP., Altamirano, ta., Rojas, N., Arellano, E., Bonacic, C.	Uso de cajas-nidos por la culebra Philodryas chamissonis (Wiegmann 1835) (Squamata, Dipsadidae) y la lagartija Liolaemus tenuis (Duméril Y Bibron 1837) (Squamata, Liolaemidae) en viñedos de Chile central. Boletín Chileno de Herpetología. 5: 29-30.
**104**	2019	San Martin-Ordenes,J., Muñoz-Leal, S., Garín, C., González-Acuña, D.	A systematic review of parasites and micropredators of non-avian reptiles (Reptilia=Sauropsida) in Chile. Zootaxa 2019, 4543, (3): 301-340.
**105**	2019	Cañas, J & Urra, F.	Philodryas chamissonis. Nocturnal activity. Herpetological review. 2019. 600
**106**	2019	Cabeza, O. Vargas, E. Ibarra, C & Urra, FA	Observations on reproduction in captivity of th endemic long-tailed snake Philodryas chamissonis (Wiegmann, 1835)(Reptilia, Squamata, Dipsadidae) from Chile. Herpetozoa 32: 203-209<br>doi: 10.3897/herpetozoa.32.e36705
